# Performance of spray‐dried *Ziziphus jujuba* extract using insoluble fraction of Persian gum–sodium alginate and whey protein: Microstructural and physicochemical attributes of micro‐ and nano‐capsules

**DOI:** 10.1002/fsn3.4081

**Published:** 2024-04-18

**Authors:** Zahra Khoshdouni Farahani, Mohamad Ebrahimzadeh Mousavi, Mahdi Seyedain Ardebili, Abdorreza Mohammadi Nafchi, Saeed Paidssari

**Affiliations:** ^1^ Department of Food Science and Technology, Faculty of Agriculture and Food Technology, Science and Research Branch Islamic Azad University Tehran Iran; ^2^ Department of Food Science, Engineering and Technology, Faculty of Agriculture and Natural Resources University of Tehran Karaj Iran; ^3^ Food Technology Division, School of Industrial Technology Universiti Sains Malaysia Penang Malaysia; ^4^ Green Biopolymer, Coatings & Packaging Cluster, School of Industrial Technology Universiti Sains Malaysia Penang Malaysia; ^5^ Department of Food Science and Technology, Damghan Branch Islamic Azad University Damghan Iran

**Keywords:** alginate biopolymer, jujube fruit, Persian gum (insoluble part), phenolic compound, spray drying, whey protein isolate

## Abstract

The study focused on the impact of the insoluble fraction of Persian gum–sodium alginate and a blend of the insoluble fraction of Persian gum–sodium alginate (IFPG‐Al) with whey protein isolate (WPI) on sprayed *Ziziphus jujuba* extract (JE) powder. The addition of whey protein led to powders with higher moisture (10%), higher solubility (99.19%), and lower powder yield (27.82%). The powders fabricated with WPI depicted the best protection of polyphenolic compounds (3933.4 mg/L) and the highest encapsulation efficiency activity (74.84%). Additionally, they had a higher *T*_g_ (62.63°C), which indicates more stability of the powders during shelf life. The sphericity of the majority of the particles was noticeable in powders, but multi‐sided concavities were visible in the protein‐containing particles. Based on the particle size's results, IFPG‐Al/WPI capsules fabricated relatively smaller particles (2.54 μm). It can be acknowledged that the presence of protein in particles can bring fruitful results by preserving valuable bioactive compounds.

## Graphical abstract

The encapsulation efficiency of *Ziziphus jujuba* extract in IFPG‐alginate/WPI powders was more (74.84%). Incorporation of WPI to IFPG‐alginate led to lower yield, higher solubility, and better protection of TPC. IFPG‐alginate capsules had more spherical particles with larger particle sizes than IFPG‐alginate/WPI.

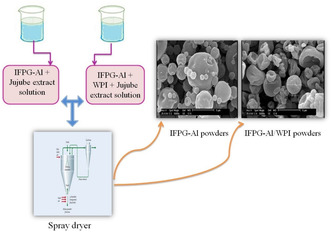

## 1 INTRODUCTION

The most common method of encapsulating environmentally sensitive compounds is known as spray drying. Its mechanism is that the compound with biological activity is covered by a suitable wall compound (Robert et al., 2010) and is sprayed and dried as a solution by the device's atomizer into the available hot space and the result is powder. The encapsulation efficiency of this method is influenced by the type of walls used (Khoshdouni Farahani, 2021). The use of single‐layer walls and multi‐layered (Khoshdouni Farahani & Khoshdouni Farahani, 2017; Sheng et al., 2021) can be noted in this method that the production of powder in the second mode is more effective (Wang et al., 2021). It is desired to apply microencapsulation to protect sensitive and bioactive compounds and use them in other food products over time (Farahani, 2021a). Fruits containing phenols, flavonoids, and antioxidants (Yang et al., 2023), such as jujubes, to protect their nutritional value and prevent the reduction of these compounds (Baltrusch et al., 2022; Khoshdouni Farahani & Mousavi, 2021) are a suitable option for micro‐encapsulation. Jujube, which is widely cultivated, especially in Iran, is one of the Rananaceae (Khoshdouni Farahani et al., 2021; San & Yildirim, 2010). Researchers have emphasized that it is an important source of antioxidants, flavonoids, and polyphenols (Badejo et al., 2020), and its valuable properties include lowering blood pressure, antibacterial, anticancer, antioxidant, and antidiabetic (Wu et al., 2011). To reduce the damage to jujube extract, a spray dryer is used to turn it into a powder (Yue et al., 2020), which is the aim of this research (Baltrusch et al., 2022; Khoshdouni Farahani et al., 2022b).

*Amygdalus scoparia Spach* (Persian gum) has two parts, soluble and insoluble, and is divided into anionic hydrocolloid (Samani et al., 2023). It is of interest due to its low price, stability, biodegradability, availability, and compatibility with the environment (Farahani, 2021c; Khoshdouni Farahani et al., 2021). Among the two parts of this gum, the insoluble fraction (IFPG) has been preferred and chosen due to its better texture and relative gel formation. During investigations, it was impossible to produce beads from Persian gum alone due to its weak and unstable structure. Therefore, due to the ability of sodium alginate to create capsules, it was used in combination with Persian gum, which can increase the productivity of native gum along with reducing costs (Khoshdouni Farahani et al., 2022a, 2023b).

One of the hydrocolloids capable of producing strong gels and capsules is sodium alginate, which can form more stable microcapsules together (Farahani et al., 2013) with other biopolymers and proteins, among which protein has considered as a compound that can form capsules and protect the core (Ghorbani et al., 2021).

Alginate, a well‐known hydrocolloid with gelling properties and suitable capsule formation, has a wide range of pH tolerance and demonstrates stronger protection by cross‐linking with other polysaccharides and proteins (Fioramonti et al., 2019) which depicted this feature in the encapsulation (by spray drying) of elderberry extract (Ribeiro et al., 2019). Baltrusch et al. (2022) also stated in their results that it is possible to produce microcapsules by spray dryer from tea extract by combining coatings of alginate and other polysaccharides. Fioramonti et al. (2019) stated that whey protein concentrate was used as a supplemental coating for spray‐drying flaxseed oil.

Whey protein, which is one of the byproducts of cheese making, causes the characteristics of film formation, coating in capsules, amphipathic, etc. (Jrad et al., 2020) and can be taken into account to produce applicable viscosity in materials and coatings with further protection (Khoshdouni Farahani et al., 2022c).

Various techniques can be used to protect extracts, particularly jujube. Efforts have been made to preserve their phenolic compounds (Di Marzio et al., 2012; Robert et al., 2010). Researchers showed that it is possible to safeguard riboflavin with hydrocolloid coatings and protein by creating beads (Wichchukit et al., 2013). Various studies have been done to confirm that alginate can make strong gels, which is the reason for the decision as a suitable option for coating (Chan et al., 2010; Kulkarni et al., 2000). In the previous studies, where other methods have been used for encapsulation using a mixture of alginate and Persian gum, the results indicated that it was fruitful and produced beads with acceptable stability (Khoshdouni Farahani et al., 2022c).

Other research has confirmed the compatibility of alginate and WPI for encapsulation (Khoshdouni Farahani et al., 2022b). In another study by Khoshdouni Farahani et al. (2023a), the encapsulation by extrusion method was performed using Persian gum (insoluble fraction) and alginate, and the results were satisfactory. Because the importance and place of phenolic products in the food basket is known and meets people's health requirements, therefore, the use of native Persian gum as a complex with alginate is followed by the spray drying method to preserve jujube compounds. Accordingly, it is important to fabricate powders with new native and available ingredients and to enrich valuable food products with them. However, the impact of the spray dryer process on the quality and characteristics of jujube powder coated with the mentioned compounds to protect and estimate the ability to increase efficiency is unclear, and it needs to be investigated in the form of this project. Therefore, the purpose of this project was to formulate carrier materials (IFPG‐Al and the combination of IFPG‐Al and WPI as carrier materials) to maintain powder attributes such as total phenol, moisture, solubility, particle size, encapsulation efficiency, yield, FTIR, SEM, and DSC as well as phenolic content of *Ziziphus jujube* extract.

## 2 MATERIALS AND METHODS

### 2.1 Materials

Whey protein isolate with 95.6% protein, Persian gum, and sodium alginate were purchased from Davisco Foods Intl., Inc., Eden Prairie, MN, USA, Sigma Aldrich (USA), and an Esfahan company, respectively. Jujube fruit was supplied from a khorasan razavi market in Iran and, finally, all reagents used were of analytical grade.

### 2.2 Preparation of extract and extraction

The fruit nucleus was removed and the remaining jujube fruits were dried at 45°C. After grinding the dried fruit, it was mixed with distilled water and shaken on a shaker at 37°C (120 rpm), and the final extract was concentrated by an evaporator (Heidolph, Laborota 4003, Germany) (Farahani, 2021b; Khoshdouni Farahani & Khoshdouni Farahani, 2017). The extract was diluted to 10° Brix (TSS) for use in samples.

### 2.3 Separation of insoluble fraction of Persian gum

An aqueous suspension (4%) prepared from Persian gum was hydrated for 48 h at 300 rpm on a shaker (25°C) (Monfared et al., 2023). After that, the solution was centrifuged for 30 min (4000 rpm) and the insoluble sediment was separated and dried in a Petri dish at a temperature of 45°C (Khoshdouni Farahani et al., 2021).

### 2.4 Carrier wall agents of spray drying

Whey protein isolate, sodium alginate, IFPG, and jujube fruit were used as main ingredients. After preparing individual solutions from them, these compounds were mixed (30 min) with jujube extract in specified concentrations and proportions until their complete combination and dissolution (Khoshdouni Farahani et al., 2024). Formulations of material combinations (Table 1) include wall materials (carriers) and *Ziziphus jujuba* extract at 600 rpm for 60 min with a stirrer and for 3 min at 12,000 rpm (Shaygannia et al., 2021) by a homogenizer device (Heidolf, Germany) before the beginning of the spray drying trial (Khoshdouni Farahani et al., 2022a).

**TABLE 1:** Encapsulation formula for *Ziziphus jujuba* extract.

Formula	Sodium alginate	IFPG	WPI	Jujube extract
Control (Pure extract)	–	–	–	10%
1	3%	5%	–	10%
2	3%	5%	16%	10%

### 2.5 Drying method

The created solutions were injected into a spray dryer (atomizer with a diameter of 0.7 mm) (DSD‐V05, Dorsa Tek, Iran) and the process conditions were constant. Jack nozzle pressure, air inlet pressure, aeration, aspiration, inlet, and outlet air temperature were 65 psi, 80 psi, 37 mbar, 70%, 140°C, and 68°C, respectively, based on the previous tests of the Spray dryer that were considered. The obtained nano and microcapsules were preserved on sealed plates until use (Khoshdouni Farahani et al., 2022a).

### 2.6 Moisture content

To assess the moisture content of jujube powders, 1 g of jujube powder at a temperature of 105°C was dried and then the weigh‐in steps were repeated to reach a constant weight (Farahani et al., 2013).

### 2.7 Solubility assessment

The powders (0.1 g) were dissolved in distilled water (10 CC) and stirred by a magnet. After the uniformity of the powder solution, its supernatant (2.5 CC) is transferred to a glass plate that has already reached a constant weight and placed in an oven temperature of 105°C (for 4.5 h). Finally, a percentage of soluble substances from the residual weight was proposed to express the solubility index (Yousefi et al., 2015).

### 2.8 Total phenolic content of *Ziziphus jujuba* extract and powders

TPCs of the resulting microcapsules based on milligrams of gallic acid equivalent per liter (dry weight) were proposed by the Folin–Ciocalteu procedure (Equation 1) (Farahani, 2021c). The amount of 750 microliters of Folin–Ciocalteu (in a ratio of 1 to 10 volume–volume) was added to the supernatant of the powders and extract (100 microliters each). Na‐carbonate solution (7.4%) was added in the amount of 300 μL after 10 min and incubated at 25°C (120 min). The standard graph was also drawn based on the concentration of 0 to 1000 mg/L of gallic acid (Yalçınçıray et al., 2020). A spectrophotometer (Cary 300, Varian, Mulgrave, Victoria, Australia) with a wavelength of 765 nm was used to measure absorbance (Asadzadeh et al., 2021).

(1) y=0.0005x+0.0714

### 2.9 Encapsulation efficiency (EE) of powder

To evaluate the encapsulation efficiency (EE) of the encapsulated extract, the method of Lupo et al. (2015) and Khoshdouni Farahani et al. (2022c) was used (Equation 2). To prepare the sample, 0.1 g of the powder was mixed with 0.1 M phosphate buffer (20 cc, 37°C) and the previous researchers' method was used to measure the total phenol until absorption at 765 nm by a spectrophotometer (Cary 300, Varian, Mulgrave, Victoria, Australia) are obtained (Thi Aenh Dao et al., 2021).

(2) $$\mathrm{EE}=\frac{\mathrm{PCP}}{\mathrm{PC}.\mathrm{PE}}\times 100$$

EE is the encapsulation efficiency, PCP is the content of phenolic compound of the powders, and PC.PE is the phenolic compounds content of the pure extract.

### 2.10 Drying yield

The relationship between the content of powder produced according to dry weight and the total content of primary solids in the solution injected into the spray dryer was used to determine the yield of spray drying (Equation 3) (Khoshdouni Farahani et al., 2022a).

(3) $$\text{Yield}=W_{\mathrm{PW}}/{W_{\mathrm{TSW}.\text{IS}}}^{*}\;100$$

where *W*_PW_ is the powder weight (g) and *W*_TSW.IS_ is the total solids weight of the injected solution.

### 2.11 Particle size distribution (PSD) and scanning electron microscopy (SEM) examination

To analyze the structure of microcapsules, a scanning electron microscope made in the Netherlands was used (SEM; Philips XL30). The powders were placed on a stub with double‐sided tape, and then a thin layer of gold was sprayed on them (Yousefi et al., 2021). The spray‐dried capsules were examined by 500×, 1000×, 2000×, and 5000× magnification (in terms of morphology).

SEM images (×2000) were surveyed by Image J software to evaluate the PSD of micro‐ and nano‐capsules. For this purpose, diameters of approximately 100 microcapsules were sized. The obtained data was reported as frequency distribution in different diameter ranges. The results were plotted in the form of a histogram to represent the curve of particle size versus frequency using Excel. The average particle size was also calculated.

### 2.12 Ft‐IR

Fourier spectroscopy (Nexus 870; Nicolet, WI, USA) was used to determine the functional groups of microcapsules. For this purpose, the wavelength range of 400 to 4000 cm^−1^ was assisted for scanning (Sirilert et al., 2018).

### 2.13 Thermal stability properties

Differential scanning calorimetry of powders was determined by module version 1.54 f (DSC 131, Setaram Instrumentation, France). Experiments were performed at an ambient temperature of 250°C (heating rate was 10°C/min) and 10 mL/min was the speed of N_2_ gas for drying (Khoshdouni Farahani et al., 2023a).

### 2.14 Statistical analysis

Variance analysis was done to check the results of all the tests with three repetitions. A significant difference (*p* < .05) was determined by Duncan and S tests (SPSS 26 software) (SPSS Inc., Chicago, IL, USA) (Nguyen et al., 2022).

## 3 RESULT AND DISCUSSION

### 3.1 Moisture content

The moisture content of powders is usually related to the drying efficiency of compounds, which is an important parameter for dehydrated materials. The higher moisture content of IFPG‐Al/WPI powder caused a significant difference compared to IFPG‐Al powder, which indicates the enhancing effect of protein on the moisture content, which is strongly influenced by the carrier (Table 2). The moisture scope of *Ziziphus jujube* extract was also within the acceptable range of fruit juice powders. In much research, the effect of various changes can be observed based on the addition of wall materials to the quantity of moisture (Khoshdouni Farahani et al., 2022a). The researchers stated that the more additional materials are used in drying, it can cause a decrease in moisture content after drying and an increase in the solid composition of the incoming feed (Fazaeli et al., 2012). The moisture content of the produced powders should be within a specified range for the stability of the powders during maintenance (Ghandi et al., 2012). Since the purpose of powder production is to preserve the primary ingredients for a longer period, therefore, less moisture causes stability over time. At the same time, powder with high moisture can have good quality. Wu et al. (2011) reported that the moisture content of the powder samples was 7.61 to 20.89%. Others also confirmed the effect of different types of carriers on moisture changes (Vidović et al., 2014).

**TABLE 2:** Total phenol of *Ziziphus jujuba* extract and spray‐dried capsules, Moisture, solubility, encapsulation efficiency, and yield of obtained powders.

	Total phenol (mg/L)	Moisture (%)	Solubility (%)	Encapsulation efficiency (%)	Yield (%)
Pure Extract	5255.2 ± 0.45^a^	–	–	–	–
Al/IFPG/JE microcapsule	2672.4 ± 0.25^c^	0.5 ± 0.10^b^	99.18 ± 0.10^a^	50.85 ± 0.50^b^	29.31 ± 0.30^a^
Al/IFPG/WPI/JE microcapsule	3933.4 ± 0.35^b^	10 ± 0.25^a^	99.19 ± 0.12^a^	74.84 ± 0.45^a^	27.82 ± 0.32^b^

*Note*: Values are mean ± SD from triplicate determinations; Different superscript letters indicate statistically significant differences among the means for each experiment (*p* < .05).

### 3.2 Drying yield

The drying yield of hydrocolloid and protein solutions is demonstrated in Table 2. Adding whey protein to the IFPG‐alginate complex affected the drying yield and caused it to decrease. When stirring the protein solution, foam is produced due to the surfactant property of proteins, and, in combination with IFPG‐alginate, a solution with a higher viscosity is created. Based on this, at the time of its injection into the dryer led to an increase in wall deposits and the performance of the dryer decreased to some extent. The yield of *Ziziphus jujuba* extract powder can vary based on the type of device, its parameters, and the type of carriers, and here it was in the range of 27.82 to 29.31%. The difference in the structure of the chemical composition of the carrier material during drying can lead to the content of different solids in the powder. Agreement of Baysan et al. (2021)'s studies with other research indicates that the performance of dried core material depends on the type of carrier used. On the other hand, other research states that the use different carriers did not make a considerable difference in the performance of powders produced from golden mulberry juice (Etzbach et al., 2020). In this regard, when producing flaxseed oil powder with whey protein along with sodium alginate, they displayed a significant performance improvement (Fioramonti et al., 2019). Carneiro et al. (2013) also acknowledged that the performance of spray‐dried products can be affected by inlet temperature, outlet temperature, feed flow rate, etc.

### 3.3 Solubility

The influence of the type of carrier on powder solubility is exhibited in Table 2. When the composition of the second protein wall (whey) was used, there was no significant change in the solubility of the powder and it showed a very slight increase. Since hydrogen bonds have the possibility of connecting to OH groups in water to create a connection with polysaccharides, hence, considerable stability can be seen in the structure of whey protein powders rather than IFPG‐alginate powder as a base carrier (Fioramonti et al., 2019). Yousefi et al. (2021) announced in their study that the addition of cellulose along with the wall material could reduce the solubility of the powder obtained from pomegranate juice. Cano‐Chauca et al. (2005) also obtained similar data from mango powder dried by spray method.

### 3.4 Total phenolic compound

Table 2 displayed that the total phenol content of IFPG‐Al/WPI powder was significantly higher than IFPG‐Al. But keeping the pure extract during the storage time causes a decrement in the phenol content, although the highest amount of polyphenol was related to it on the initial day, and this has also been confirmed in the studies of others (Khoshdouni Farahani et al., 2022a). According to these results, the production of powder to protect the phenolic compounds of *Ziziphus jujuba* extract by spray drier provides the basis for longer shelf life. The total phenol of sprayed microcapsules was significantly different from *Ziziphus jujuba* extract (*p* < .05). This could be due to inadequate extraction of polyphenols from the particles and also due to their wastage during spray drying. But over time, the pure extract will reduce significantly according to other studies (Khoshdouni Farahani et al., 2022c). The amount of polyphenols is also changed based on the type of carrier, which has been explained in other reviews. It has been proven that the amount of polyphenol in *Ziziphus jujuba* extract decreases due to some reasons during the drying process, which include oxygen, conditions of process performance, high temperature, etc. (Kuck & Noreña, 2016). These researchers have pointed out that wall‐coating compounds significantly affect the stability of polyphenols in this technique. The amount of polyphenols in IFPG‐Al and IFPG‐Al/WPI powders were 2672.4 and 3933.4 mg of gallic acid per liter, respectively. The combination of two Persian (IF) and alginate hydrocolloids led to the extraction of more polyphenols, which was because of the decrease in the hardness of the gel structure due to the less alginate in the capsules (half of it), since it was shown in Khoshdouni Farahani et al. (2022a, 2022b, 2022c) research shows that when the alginate matrix is used in the entire capsule structure, the amount of phenol extraction from capsules is less (2081.2 to 2949.2 mg gallic acid/L). Li and Wood (2019) acknowledged that the presence of whey protein can protect the compounds of the capsules from oxidation since it has the effects of creating an emulsion and the IFPG‐Al/WPI treatment showed the highest amount of phenolic compounds in the present work.

### 3.5 Encapsulation efficiency

The importance of the constancy of powders is determined based on the encapsulation efficiency (EE), which is affected by the variety of coating materials (Table 2). The encapsulation efficiency of jujube extract powder obtained from a spray dryer was between 50.85 and 74.84%. There was a minimum amount of polyphenols in IFPG‐Al powder, and a more extensive protective effect of IFPG‐Al was seen in binding with whey protein, which naturally also showed in efficiency (Markakis et al., 2015). It has been determined that the formation of film and capsule is one of the characteristics of wheat powder. Hereupon, using it in the microcapsules obtained from the powder augments the encapsulation performance (Khoshdouni Farahani et al., 2022a, 2022b, 2022c).

The effect of greater consistency because of the higher viscosity of the injection solution due to the nature and the presence of protein in the dryer caused higher retention of polyphenols. Due to the existence of a direct relationship between polyphenols and efficiency, the EE of the whey‐based powder was also higher. Based on the significant results obtained, which were mainly seen in the efficiency tests, the amount of polyphenols, etc. of the powders, which is due to the discrepancy in coating materials, IFPG‐Al/WPI powder is preferred for *Ziziphus jujuba* extract protection. Other researchers have also confirmed that creating a link between protein and hydrocolloids can lead to better preservation of *L. delbrueckii* bacteria in the internal pathway of the body (Shinde et al., 2014). Anthocyanins (existing in pomegranates), which are types of polyphenols, showed significantly higher encapsulation efficiency when protected by coatings along with soy protein than the original encapsulation material (Robert et al., 2010). Resembling results were also reported which confirmed the present study (Tumbas Šaponjac et al., 2016). Khoshdouni Farahani et al. (2022c) reported the encapsulation efficiency of jujube extract with alginate by extrusion method between 6.70% and 25.85%, which is much lower than the present method. Also, in another study, (Khoshdouni Farahani et al., 2023a) stated the efficiency of encapsulation using alginate and pea protein was between 8.47 and 11.65%.

### 3.6 Morphology and size distribution of capsules of powders

**FIGURE 1:**
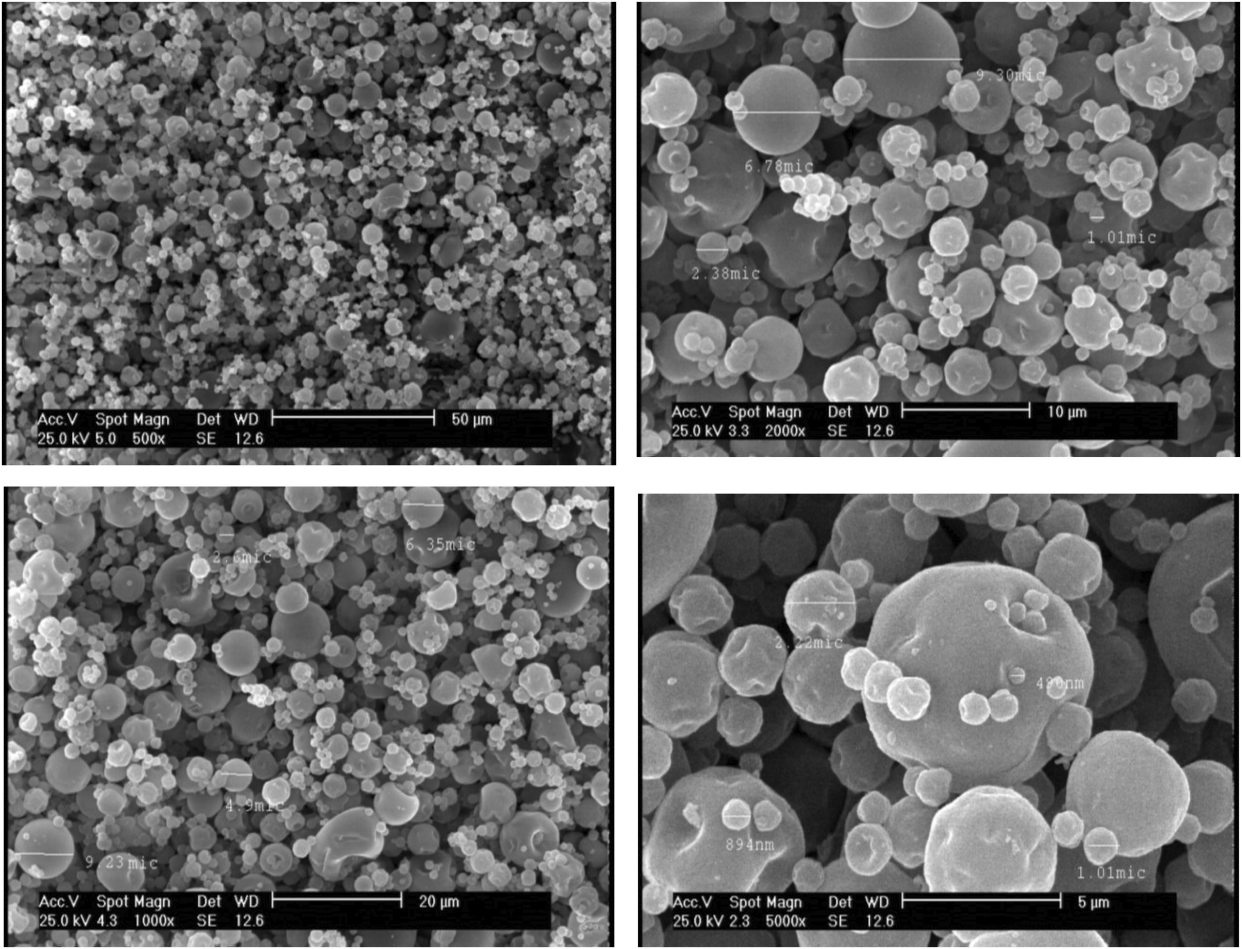
Scanning electron microscopy images of spray‐dried insoluble fraction of Persian gum–sodium alginate/*Ziziphus jujuba* extract capsules at 500×, 1000×, 2000×, and 5000× magnification.

**FIGURE 2:**
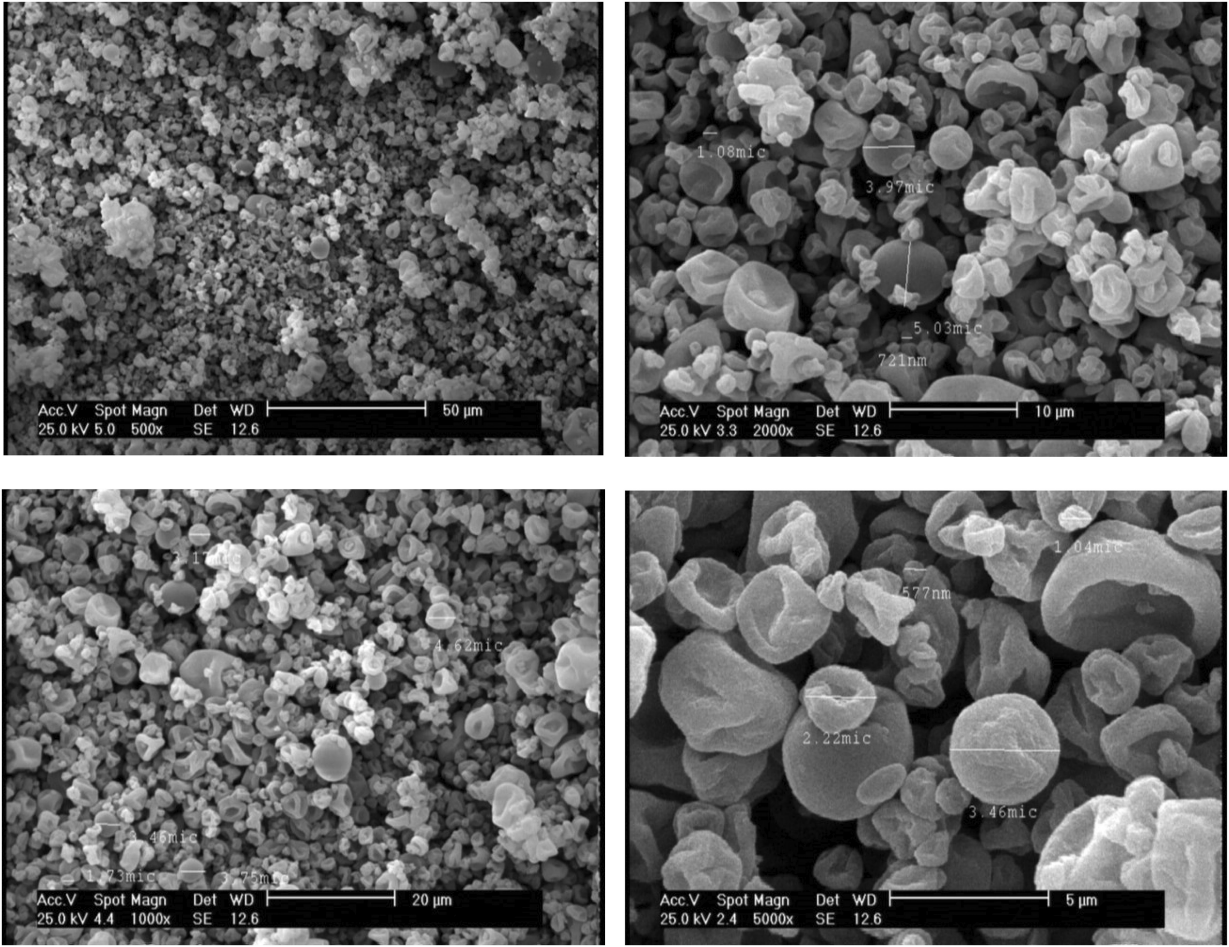
Scanning electron microscopy images of spray‐dried insoluble fraction of Persian gum–sodium alginate/WPI/*Ziziphus jujuba* extract capsules at 500×, 1000×, 2000×, and 5000× magnification.

Core–carrier interactions and chemical attributes can affect the drying conditions of injectable solutions during the spray operation. The drying performance is influenced by parameters such as the drying conditions of the device, the evaporation rate of the dissolved solvent, the size of the particles, etc., which change the morphology of the produced particles under its action, as well as, air inlet pressure can change the particle size. SEM images of spray‐dried jujube microcapsules are demonstrated in Figures 1 and 2. The distinction in the structure of various coating materials proved that the microstructure of microcapsules can be set by the reactions that occur between the central layer and the coatings (Guzar, 2012).

The outcome of the capsule dispersion indicates the sphericity of the majority of powder particles and the effect of the pressure created by the pump has caused the creation of capsules with multi‐sided indentations to a lesser extent. The surface indentations in the images of the powders were significantly higher in IFPG‐Al/WPI, which is reported to be a common occurrence in alginate‐containing treatments (Chaumun et al., 2020). There were also wrinkles and shrinkage in some IFPG‐Al/WPI capsules. There is a possibility of loss of some extract content in several IFPG‐Al/WPI capsules due to the great indentations in them. The size distribution of the capsules is such that microcapsules were in the majority and nanocapsules were in the minority. The high temperature in the heat chamber can cause an appearance in the form of semi‐collapse on the particle surfaces and rapid removal of water content (Shaddel et al., 2018).

**FIGURE 3:**
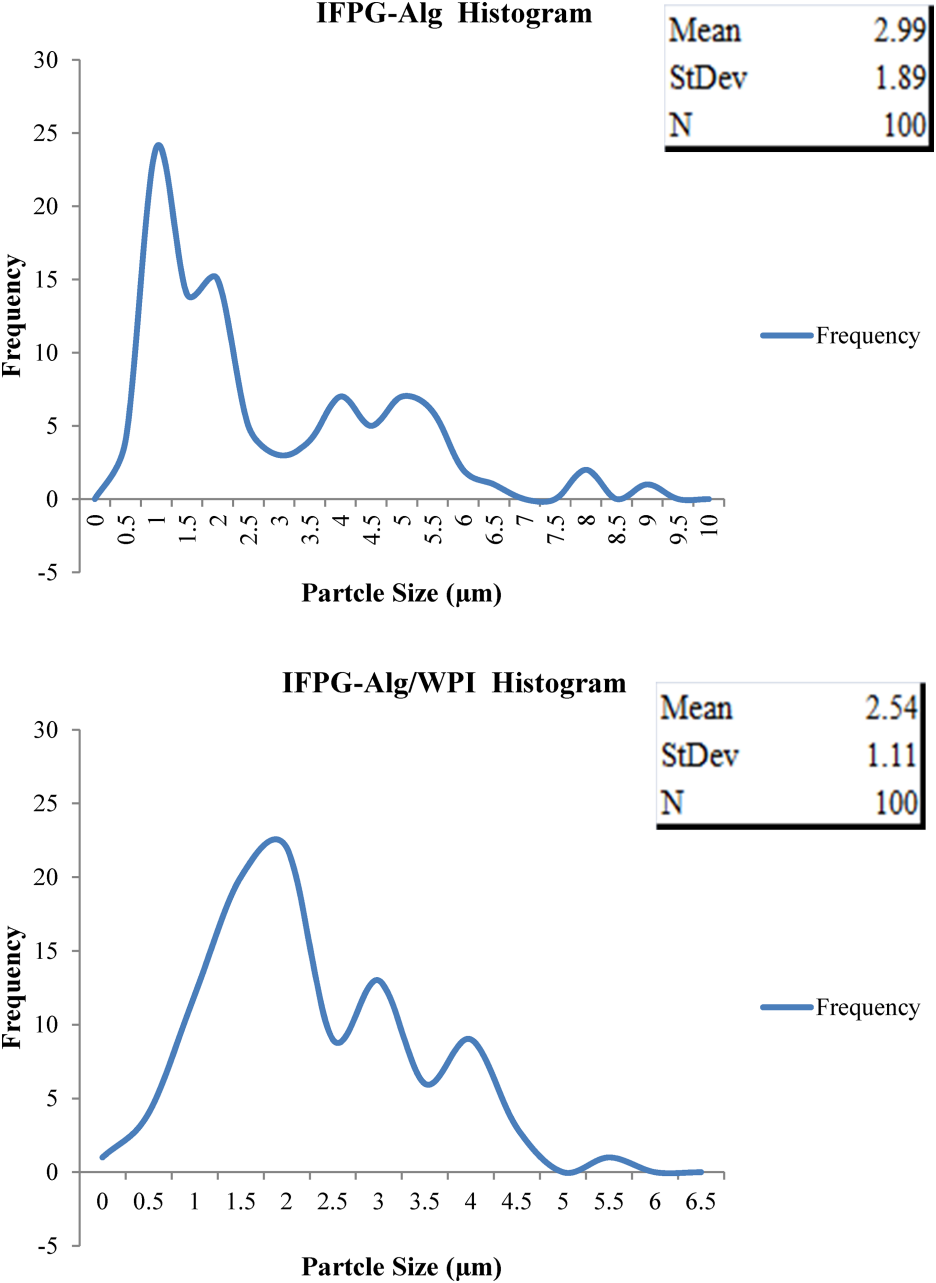
Particle size distribution of insoluble fraction of Persian gum—sodium alginate/*Ziziphus jujuba* extract and insoluble fraction of Persian gum–sodium alginate/WPI/*Ziziphus jujuba* extract capsules, by frequency (at 2000×).

Figure 3 can be presented the size distribution of IFPG‐Al and IFPG‐Al/WPI particles. The results indicate that the particles of IFPG‐Al capsules have an average size of 2.99 μm, but IFPG‐Al/WPI capsules produced relatively smaller particles of size 2.54 μm, which is due to more potholes and shrinkage and the loss of spherical structure which has caused a slight decrease in the size of the particles. Although the structure of the IFPG‐Al/WPI capsules is slightly wrinkled and accumulated, due to the initial viscosity of the solution, the protection of phenols is still higher and only exhibits more protection than IFPG‐Al capsules. However, the IFPG‐Al particles exhibited a slightly larger average particle size, with the range of 1.4 and 2.3 μm reported as the most particle size distribution, and reaching the peak at 1.4 μm. Following this, the PSD of IFPG‐alginate/WPI (2.2 and 3.3 μm with more than ten repetitions) peaked at about 2.2 μm, which, its peak point was almost 1.5 times that of IFPG‐Al particles. Fioramonti et al. (2019) in their study of carriers found that protein samples show larger capsules that are sometimes due to the two‐layer wall and the accumulation of large alginate molecules that cause bridges. In the present study, the more uniformity of the size of the IFPG‐alginate/WPI particles, and their excessive shrinkage has led to the display of less particle size.

### 3.7 FTIR of *Ziziphus jujuba* powders

**FIGURE 4:**
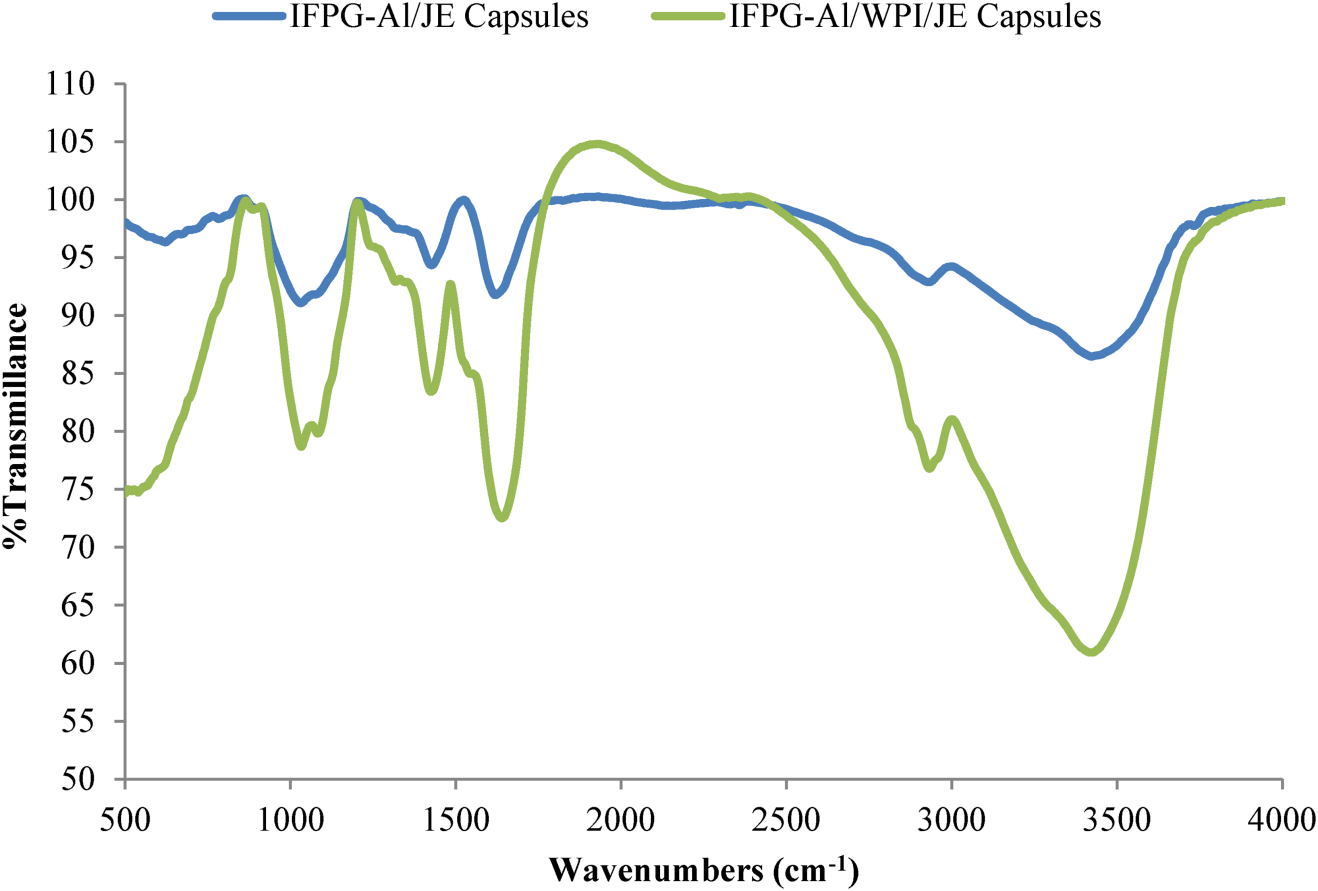
Infrared spectroscopy (FT‐IR) images of spray‐dried capsules; insoluble fraction of Persian gum–sodium alginate/JE and insoluble fraction of Persian gum–sodium alginate/WPI/JE capsules.

Figure 4 demonstrates the wave numbers of spray‐dried *Ziziphus jujuba* powders. Carboxylic vibration of alginate structural groups is present at 1421–1425 cm^−1^ and its hydrogen groups at 1341 cm^−1^. The wavenumber of 1601 cm^−1^ displayed the symmetric carboxylic stretching vibrations in alginate. The ionic salt shows a wave number of 1015 cm^−1^ due to the C‐O group. Belscak‐Cvitanovic et al. (2015), Peanparkdee et al. (2016) and Khoshdouni Farahani et al. (2022c) also confirmed that the polyphenols in the extract can display the wave number of 3421.75 cm^−1^ which is caused by hydrogen groups. Whey proteins depict peaks in the range 1522, 1639.92, and 1531 to 1571, which correspond to aggregation (molecular β‐sheets), asymmetric β‐sheets, and unfolded polypeptide fragments, respectively. The wave number at 1448–1427 cm^−1^ is related to Persian gum, as stated by the researchers, and shows the carboxylic groups or vibration of CH_3_ links (Emamverdian et al., 2020). In another survey, the polysaccharide peaks of Persian gum indicated the CH_2_ and vibration of hydroxyl bond at wave numbers of 2927 and 3424 cm^−1^, respectively (Gharanjig et al., 2020).

### 3.8 Thermal stability of powders (DSC)

**FIGURE 5:**
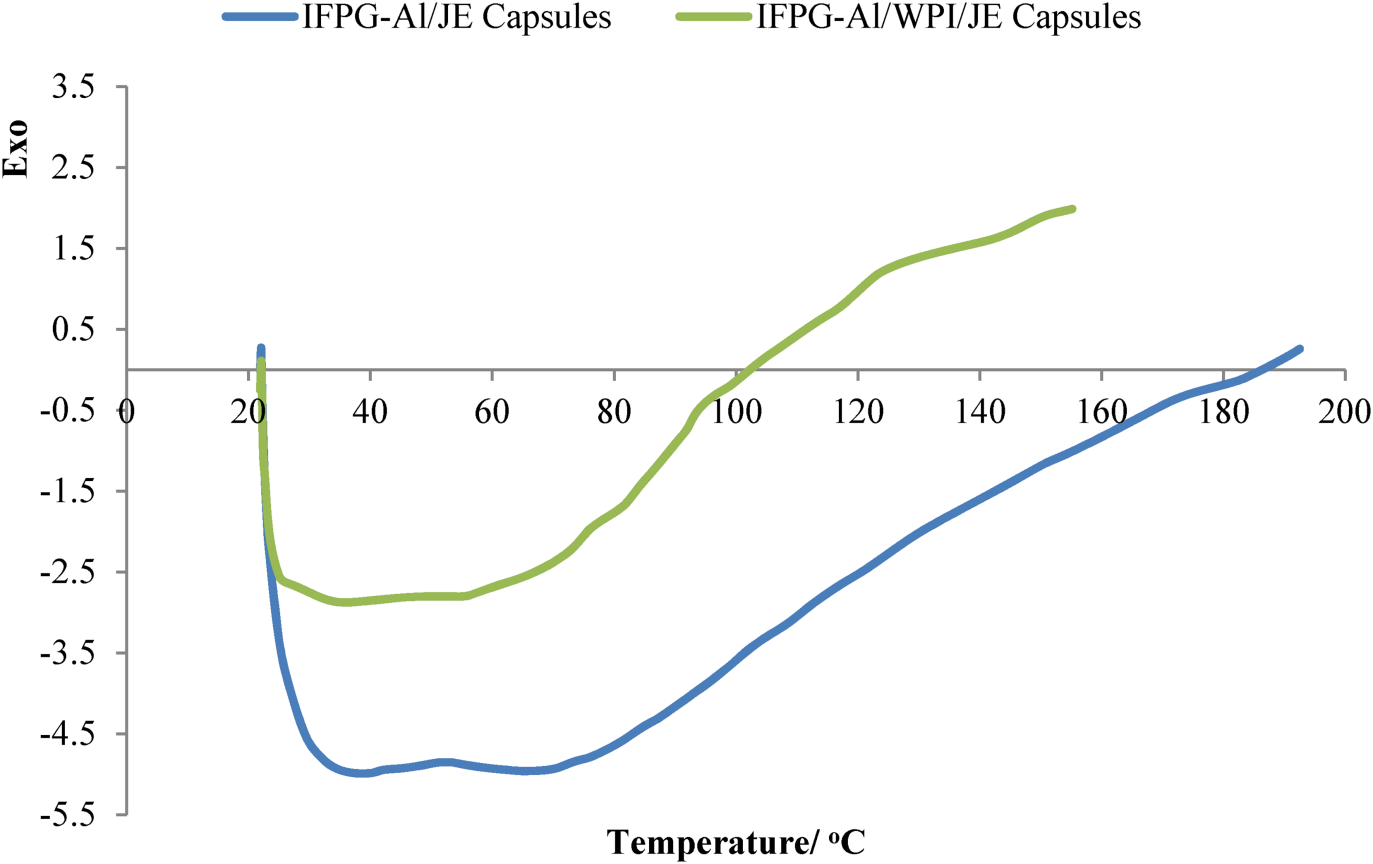
DSC spectra of spray‐dried capsules; insoluble fraction of Persian gum–sodium alginate/JE and insoluble fraction of Persian gum–sodium alginate/WPI/JE capsules.

DSC plots of spray‐dried *Ziziphus jujuba* extract with IFPG‐Al and IFPG‐Al/WPI are depicted in Figure 5. Comparison of the curves obtained from different formulations of materials indicates different thermal changes of different species of powders (Tonon et al., 2009). *T*_g_ temperatures for IFPG‐Al and IFPG‐Al/WPI treatments were 42.49°C and 62.63°C, respectively. When the glass transition temperature is higher, it indicates that the powders are more stable during the shelf life, which increased this temperature by about 20°C when adding protein in this process. After that, the endothermic peak was shown at 55.17°C and 61.23°C for IFPG‐Al and IFPG‐Al/WPI powders, respectively.

Córdoba et al. (2013) stated that the presence of interaction among polymers and polyphenols causes an increment in temperature in extract capsules. Also, Endothermic peaks are relevant to hydrophilic compounds in biopolymers. The amount of polyphenols confirms the fact that higher glass transition temperature in IFPG‐Al/WPI powder leads to greater preservation of the extract. The researchers mentioned in their project that the cocoa extract encapsulated with alginate exhibits an endothermic peak (caused by the binding between polyphenol and sodium alginate), which is a confirmation of this study. An increase in the glass transition temperature of the microcapsules is also visible (effect of molecular hydroxyl group bonding by polyphenol) (Lupo et al., 2015). The first visible endothermic peaks usually indicate structural variations caused by heating proteins (Swain et al., 2005). The existence of structural interactions (between proteins and polysaccharides) is one of the factors of creating more thermal stability in capsules. In addition to these cases, an exothermic peak can indicate the melting point (Fontes et al., 2013).

## 4 CONCLUSION

Various wall materials can have an extensive and different impact on dried powders and their structural and chemical quality. The results of this study acknowledged that the highest efficiency of powder encapsulation was achieved when the combination of IFPG and sodium alginate carriers was available with WPI. Microcapsules with protein walls were able to display a higher quantity of phenolic compounds and larger particle size, which in addition to improving the physicochemical characteristics, can be said that the presence of protein also increases the nutritional value of the resulting powder. However, it can be used through this way from the waste of cheese factories in such products. Besides reducing costs in the production of bioactive powders, these combined carriers with native hydrocolloids can also be used for the preparation of functional food products and the extracts rich in polyphenols can be delivered to consumers in the form of products containing these micro‐ and nano‐powder capsules.

## AUTHOR CONTRIBUTIONS

**Zahra Khoshdouni Farahani:** Conceptualization (equal); data curation (equal); formal analysis (equal); funding acquisition (equal); investigation (equal); methodology (equal); resources (equal); software (equal); visualization (equal); writing – original draft (equal). **Mohamad Ebrahimzadeh Mousavi:** Project administration (equal); validation (equal); visualization (equal); writing – review and editing (equal). **Mahdi Seyedain Ardebili:** Project administration (equal); supervision (equal); validation (equal). **Abdorreza Mohammadi Nafchi:** Writing – review and editing (equal). **Saeed Paidssari:** Software (equal).

## FUNDING INFORMATION

This research did not receive any specific grant from funding agencies in the public, commercial, or not‐for‐profit sectors.

## CONFLICT OF INTEREST STATEMENT

The authors declare that they have no known competing financial interests or personal relationships that could have appeared to influence the work reported in this paper.

## Data Availability

The data may be available from the corresponding author upon reasonable request.
